# Intraoperative irradiation: precision medicine for quality cancer control promotion

**DOI:** 10.1186/s13014-017-0764-5

**Published:** 2017-02-02

**Authors:** Felipe A. Calvo

**Affiliations:** 1 Department of Radiation Oncology, Department of Oncology, Hospital general Universitario Gregorio Marañon, Complutense University of Madrid, Madrid, Spain; 2 Instituto de Investigación Sanitaria Gregorio Marañon, Grupo Oncologia Interdisciplinar y Biotecnológica. Proyecto PI15/02121, Madrid, Spain; 3 Instituto de Salud Carlos III. Ministerio de Economía y Competitividad. Gobierno de España, Madrid, Spain

**Keywords:** Intraoperative irradiation, Precision medicine, Real-time surgical exploration, High single dose radiotherapy

## Abstract

Intraoperative irradiation was implemented 4 decades ago, pioneering the efforts to improve precision in local cancer therapy by combining real-time surgical exploration/resection with high single dose radiotherapy (Gunderson et al., Intraoperative irradiation: techniques and results, 2011). Clinical and technical developments have led to very precise radiation dose deposit. The ability to deliver a very precise dose of radiation is an essential element of contemporary multidisciplinary individualized oncology.

This issue of *Radiation Oncology* contains a collection of expert review articles and updates with relevant data regarding intraoperative radiotherapy. Technology, physics, biology of single dose and clinical results in a variety of cancer sites and histologies are described and analyzed. The state of the art for advanced cancer care through medical innovation opens a significant opportunity for individualize cancer management across a broad spectrum of clinical practice. The advantage for tailoring diagnostic and treatment decisions in an individualized fashion will translate into precise medical treatment.

## Precision medicine

Precision medicine refers to “*prevention and treatment strategies that take individual variability into account”* [1]. It is a fortunate terminology to describe the recent evolution of clinical medicine based upon the contribution of translational research from basic biomedical sciences (“omics”) into modern daily practice. Precision oncology adapts this integral clinical vision to an individualized (personalized) care to cancer patients [2]. Both cancer and patients are bio-heterogeneous and their medical approach requires precise decisions selected on the basis of the bio-profile of “that cancer” and “that patient”. At the present time, the dominant interpretation in oncology generally contains a reductionist argument in which precise medicine is, in practice, limited to a select group of pharmaceutical compounds for specific bio-targets detected in specific cancer mutations [3, 4].

Precision medicine in oncology has been applied in clinical practice for decades. The better understanding of cellular, molecular and sub-molecular diversity, in conjunction with improved interpretation of patient biometrics (age, comorbidity, social conditions, etc.), has led to a progressive refinement in precision oncology.

Oncology practice in the 21st century requires a wide and strong consensus of coordinated interdisciplinary character including established guidelines for the integral management of complex oncologic diseases. Precision oncology today should be a continuum of care across multiple specialties creating a transverse reality requiring updated precision medicine in each interdisciplinary component of clinical practice, including diagnostic and therapeutic decisions [5]. Transverse care includes the best radiobiology knowledge, state of the art clinical and diagnostic techniques and therapeutic innovation. Outside the committed team-culture of Multidisciplinary Tumor Board (MTB), evidence-based or innovated (evidence-generating) excellent clinical practice should be the goal of every oncology team. Precision oncology in up-to-date surgical, medical and oncology care requires MTB enrichment [6].

Local therapy for cancer control is an imperative requirement (*conditio sine qua non*) for disease-free and overall survival. There is sufficient epidemiologic evidence that there is an improvement in cancer control in large national and international population-based registries including specific cancer cohorts (breast, colorectal, etc.), as well as in cancer as disease diagnosis [7]. The contribution of improved local treatment to survival is well documented in the breast cancer model (localized disease) [8, 9] and an equivalent effect should be expected in human cancer subtypes with similar bio-patterns of control/recurrence and cure (based on the risk of systemic progression). Precision in local therapy benefits both from improved target definition and normal tissue protection. Intraoperative irradiation is vision guided, fingers (tactile) guided and surgically guided radiotherapy (SGRT): it is, at the present time, the best possible guided real-time irradiation technique. In large anatomical cavities (abdomen, pelvis, thorax) the direct access of irradiation to the cancer containing surgical bed (or unresected lesion) at the time of exploration/resection allows to displace and mechanically protect numerous dose-sensitive normal tissues uninvolved by cancer, like: ureters, bladder, vagina, uterus, prostate, rectal stump, small bowel, colon, stomach, kidney, bile duct, pancreatic remnant, esophagus, lung, skin, chest wall etc. It is an obvious benefit from the opportunity of real-time radiosurgical collaboration, but could be undervalued: it is an extremely valuable version of precise radiation oncology technology, characteristic for intraoperative irradiation, decisive for long-term survivors and their normal tissues.

## Technology: dosimetric diversity implies a non-equivalent clinical results scenario

Equipment developed for intraoperative clinical use of ionizing radiation includes isotopes (brachytherapy), low energy kilovolt X-rays generators and high energy electron beam linear accelerators. A global tendency to miniaturize the technical elements for radiation delivery is evident in the last 2 decades. The promotion of intraoperative irradiation in a hospital-based cancer program requires a level of adaptation to the complexity of a surgical suit and hospital structure. Dose deposit characteristics has radical differences among available technologies: dosimetry performance shows a step dose-gradient in brachytherapy and Kv delivery (together with a single useful energy available), while high energy electron accelerators provide the opportunity to select among a variety of homogeneous dose distributions, able to treat desired thickness of targets (tissues at risk of cancer) depending upon the electron beam energy elected. The 3D geographical target coverage is also much different upon technologies mentioned. The institutional experience at the General University Hospital Gregorio Marañon in Madrid (Spain) reported a total of 1004 patients treated in the period 1995–2012. The use of electron energies ranged from 6 MeV to 18 MeV (78% 12 MeV or less). Applicator diameter ranged from 5 to 15 cm 75% beveled 15–45°, 9% using multiple field arrangements. In our experience, rectal, pancreatic, gastro-esophageal cancer and soft tissue sarcomas benefited in practice, individualized criteria from the availability of abundant options of geographic and dosimetric configurations [10]. A recent analysis of clinical practice adaptation to a dedicated miniaturized linear accelerator found that in the same institution in 79 procedures (period December 2013 to July 2014) for breast, pancreas, rectal cancer, oligo-recurrences and soft tissue sarcomas, 112 different configurations (energy, size and beveled end combinations) were required for treatment [11].

The relevant factors to guide dosimetric decisions in intraoperative irradiation are high dependent of intra-surgical conditions of the bio-target to be treated. Uncertainties such as fluid instability, irregular non-uniform surgical post-resected surface, air-gap and tissue displacement during intraoperative radiation delivery, are properly compensated by the use of dedicated linear accelerators with high energy and high dose-rate electron beams.

## Radiobiology: intensification vs sub-intensification

Precision in dose-deposit allows to explore the use of single high dose irradiation, even more in the context of normal tissue protection by mechanical displacement. Tolerance of normal tissues in large animal models have been meticulously explored using single escalated doses (range 10 to 40 Gy) or escalated boost levels (10 to 30 Gy) combined with a component of normo- fractionated external beam radiotherapy (50 Gy). Human data recommends 20–25 Gy single dose or 10–15 Gy boost dose plus 50 Gy as efficient cancer control schemes for in a variety of cancer sites and histological subtypes [12]. Cellular and sub-cellular models are of value for potential modulation of radiation effects in the intra-surgical scenario (low cancer cell burden, high content in growth factors and repair/inflammatory reactions), but the long-term clinical therapeutic-index data is already available. A level of prediction in cancer control has been recently reported in rescued recurrent sarcoma patients using the EQD2 model (survival benefit for EQD2 > 62 Gy) [13]. Re-analysis of the available clinical information introducing biological elements of discrimination (BED, EQD2, molecular profiles, etc) will generate new hypothesis to individualized practice from retrospective (large and mature) data sets.

A unique experience using exclusive 21 Gy post-tumorectomy electron irradiation in localized breast cancer has provided pioneering information of clinical correlations among bio-risk and local cancer control [14]. In >1800 patients treated at the European Institute of Oncology age, tumor size, nodal and cerb-2 status and perineural invasion, significantly impacted local control. Re-analysis of the data according to ASTRO and ESTRO recommendations for accelerated partial breast irradiation (APBI), patients in the most favorable category (suitable) had a 1.5% incidence of local recurrence. Recently, ASTRO considers the available data with 21 Gy electron intraoperative irradiation recommendable for practice in the best prognostic APBI category with a 100% agreement among experts involved in the 2016 update criteria [15].

## Polyvalent clinical results in cancer: the supremacy of electron beam data

This issue of *Radiation Oncology* contains an extensive elaboration of clinical results in genitourinary (47 studies, >3100 patients), breast (6 studies, >1500 patients), rectal (11 studies, >1700 patients) pancreatic cancer (13 studies, >1500 patients) and soft tissue sarcomas (22 studies, >1700 patients) treated with dose-radiation-escalation strategies and surgery. Over 90% of the data corresponds to electron intraoperative irradiation approaches, while brachytherapy is present in a minority of genitourinary and sarcoma publications, and Kv in the early breast cancer model. The electron references show consistent and reproducible results in terms of local control when a boost component is delivered intraoperatively for cancer sites requiring multimodal therapy. It is relevant to emphasize that escalated radiotherapy promotes high local control rates in R0, R1 and R2 resection scenarios including extended surgery for oligo-recurrent disease. For the unfavorable resection status (R+) boosting with intraoperative electrons has proven to be superior to external beam radiotherapy alone in rectal [16], pancreas [17] and oligo-recurrent cancer [18, 19], while moderate boost dose-escalation >12.5 Gy improves local control in extremity soft tissue sarcomas [20]. Additionally, electron-based intraoperative irradiation have reported high local control rates in pediatric cancer including sarcomas and oligometastatic disease [21, 22].

A bibliometric analysis [23] of PubMed available publications in the period 1997–2013 using science citation index impact factor as a quality indicator showed 972 papers on intraoperative irradiation topics, 41% in surgical journals, being cancer clinical outcome the most frequently published primary topic (*n* = 661, 68%; *p* < 0.001). The median impact factor significantly improved with time: 2.396 in the period 1997–2001 to 3.798 in 2007–2012 (*p* = 0.006).

There is a large and mature body of evidence supporting the feasibility, tolerance and cancer control promotion using intraoperative radiotherapy as a component of treatment in patients requiring surgery and radiotherapy as evidence-based strategy. The recommendation of randomized trials to establish improved cancer management testing radiation technology innovations is methodologically questionable [24] and contradictory with individualized-personalized cancer health-care precision medicine era.

## Research and innovation: precision for quality and safety

Biomedical engineering cooperation is capital for technological innovation and research is the motor of knowledge [25]. Intraoperative irradiation has active programs to implement adapted simulation and treatment planning systems [26], alternatives to further optimize planning by the use of intraoperative imaging [27], control of dose-deposit in the clinical target in real-time, which has been tested with success with in vivo dosimetry instruments for measuring electron beam procedures [28–30] and risk models to analyze and estimate potential errors in a complex multi-professional coordinated activity such as intraoperative irradiation [31].

Laparoscopic surgery (locally advanced rectal cancer model) is compatible with electron-based intraoperative irradiation [32]. Surgical navigation has been explored in large abdominal open surgical procedures by the adaptation of the surgical suit to a stereotactic controlled space with optical cameras and references set in the patient anatomy and in the electron beam applicator [33]. Progress in imaging will potentiate pre-planning, intra-planning, guidance of radio-surgical maneuvers, registration and documentation of technical parameters.

Teaching, education and training are key elements for quality practice implementation improving the learning curve in new programs or the re-adapting techniques in expert institutions. New initiatives are launched for sharing knowledge in clinical-case solving problems oriented websites [34] or as expert-based task forces under the umbrella of a scientific society (ESTRO IORT Task Force) to generate guidelines for quality assurance, updated and safe clinical practice [35].

Future development of intraoperative irradiation will be built on the basis of precision medicine and personalized oncology. Technological developments are already here (as work-in-progress) to further improve super-precision in terms of dose-delivery, dose-measurement, dose-registration and dose-integration with other treatment factors (radiation and surgical integral scenario). Biological models of interest to be explored include the identification of radio-resistant cancer profiles and metastatic risk (incorporating early assessment of circulating tumor cells). Clinical heterogeneity can be structured into nomograms [36] for individualized risk-adapted treatment recommendation including the intraoperative irradiation component. NCCN has incorporated IORT components in several cancer sites in its 2016 version (http://www.nccn.org).
